# Do we still need IQ-scores? Misleading interpretations of neurocognitive outcome in pediatric patients with medulloblastoma: a retrospective study

**DOI:** 10.1007/s11060-017-2582-x

**Published:** 2017-08-04

**Authors:** Barbara Wegenschimmel, Ulrike Leiss, Michaela Veigl, Verena Rosenmayr, Anton Formann, Irene Slavc, Thomas Pletschko

**Affiliations:** 1 Department of Pediatrics and Adolescent Medicine, Medical University of Vienna, Waehringer Guertel 18-20, Vienna, Austria; 2 Association for the Promotion of Childhood and Adolescent Neurology, Psychiatry, Psychology and Psychotherapy (kjnp3), Vienna, Austria; 3 Neuropsychology Unit, Department of Neurology, University Hospital Zurich, Zurich, Switzerland

**Keywords:** Oncology, Medulloblastoma, IQ, Intelligence, Neurocognitive functions, Information processing speed

## Abstract

Over the past decades, many studies used global outcome measures like the IQ when reporting cognitive outcome of pediatric brain tumor patients, assuming that intelligence is a singular and homogeneous construct. In contrast, especially in clinical neuropsychology, the assessment and interpretation of distinct neurocognitive domains emerged as standard. By definition, the full scale IQ (FIQ) is a score attempting to measure intelligence. It is established by calculating the average performance of a number of subtests. Therefore, FIQ depends on the subtests that are used and the influence neurocognitive functions have on these performances. Consequently, the present study investigated the impact of neuropsychological domains on the singular “g-factor” concept and analysed the consequences for interpretation of clinical outcome. The sample consisted of 37 pediatric patients with medulloblastoma, assessed 0–3 years after diagnosis with the Wechsler Intelligence Scales. Information processing speed and visuomotor function were measured by the Trailmaking Test, Form A. Our findings indicate that FIQ was considerably impacted by processing speed and visuomotor coordination, which leaded to an underestimation of the general cognitive performance of many patients. One year after diagnosis, when patients showed the largest norm-deviation, this effect seemed to be at its peak. As already recommended in international guidelines, a comprehensive neuropsychological test battery is necessary to fully understand cognitive outcome. If IQ-tests are used, a detailed subtest analysis with respect to the impact of processing speed seems essential. Otherwise patients may be at risk for wrong decision making, especially in educational guidance.

**Electronic supplementary material:**

The online version of this article (doi:10.1007/s11060-017-2582-x) contains supplementary material, which is available to authorized users.

## Introduction

Over the past decades, many studies on neurocognitive outcome of children who suffered from a Central Nervous System (CNS)-tumor or other forms of pediatric cancer predominantly reported the full scale intelligence quotient (FIQ)—often referred to as “g-factor”—as an indicator of the patients’ intellectual performance [1–3]. Moreover, due to a lack of standardized neuropsychological tests for children and adolescents (especially in the past), a lack of time, knowledgeable staff or an insufficient reimbursement of costs [4], in many pediatric oncology centers, solely IQ-tests are used for neurocognitive monitoring instead of a standard of care neuropsychological test-battery. Actually, this screening practice has to be put into question since it is well known that pediatric cancer survivors, especially CNS-tumor survivors, are at a higher risk for declines in some of their cognitive abilities (e.g. information processing speed) in the course of the illness [5]. Consequently, they either achieve fewer developmental milestones or achieve them when they are older than their healthy peers [6]. However, the FIQ is possibly not the correct indicator of a decline, especially taking international guidelines about neurocognitive screening and monitoring into account [4, 7, 8]. Nevertheless, for a long time studies on cognitive late effects solely used the FIQ to draw conclusions about a patient’s general cognitive ability and his/her further academic achievement [1, 2, 9–11]. Some of these approaches distinguish between verbal IQ (VIQ) and performance IQ (PIQ), indicating that FIQ consists of at least two components. More recent studies started to investigate long-term outcome in detail, including neurocognitive domains like information processing speed [3, 12–16]. However, despite the suggestions of the mentioned guidelines [7, 8], the impact of these neurocognitive functions on the calculation of IQ-scores is still largely neglected. Still, the FIQ is quite commonly used for decision making in educational guidance, ignoring the large amount of research on specific neuropsychological dimensions and how they relate to academic performance.

It has to be noted that, technically, the FIQ is only a composite score, which is not able to measure the extent of cognitive impairment in single domains. The FIQ is based on a compensation model, i.e. a deficit in one subtest (e.g. logical reasoning) can be compensated by a better performance in another subtest (e.g. vocabulary) [17]. Therefore, the FIQ is likely to underestimate these impairments [5]. Moreover, it is obvious that the FIQ can vary significantly within one person, depending on the definition of intelligence (e.g. some tests solely use logical reasoning as indicator of the g-factor), as well as on tests and subtests that are used for assessment (e.g. some subtests have a time limit for each item, others not; some subtests require visuomotor functions, others require verbal functions). A commonly used subtest is the subtest “block design” of the Wechsler Intelligence Scales (WS), where patients have to rearrange blocks with their hands according to a given pattern (visuomotor coordination). The faster this rearrangement takes place, the more points are awarded (processing speed). This is true for older and newer versions of the WS.

Information processing speed is actually a basic cognitive function reflecting the speed at which a person can process a cognitive task [18, 19]. Hence, slower processing speed or poor visuomotor function may lead to declines in FIQ as described above. When speed is impaired by the tumor or its treatment, e.g. irradiation [19], it is, consequently, inaccurate to conclude a general cognitive decline (since other functions may not be affected).

In summary, this study is targeted at comparing the FIQ-approach and the “neurocognitive approach”. This paper especially focuses on the influence of two single neurocognitive functions, processing speed and visuomotor function, on the general cognitive performance (FIQ) of children with medulloblastoma (MB). These two domains were chosen, since research has shown that patients with brain tumors after craniospinal irradiation are at high risk for a “slowing down” of cognitive abilities [3, 5], that poor visuomotor coordination is directly related to lesions of the cerebellum [20], and processing speed and visuomotor coordination are functions needed in several subtests of the WS, which are worldwide commonly used.

### Objectives

In order to address the above mentioned issues, we formulated two specific research questions.

1. In which way do FIQ, VIQ and PIQ, as well as the mean subtest values in intelligence tests of pediatric patients with MB differ from the normative sample? Is there a higher deviation from the norm in those subscales which include a speed- and/or visuomotor-component?
2. Is there an influence of processing speed/visuomotor function on IQ-scores, i.e. is there a significant difference in IQ-scores between patients with at least average processing speed/visuomotor function scores and patients with a performance below average?

## Patients and methods

### Measures

To analyse our research questions, we retrospectively used data from the neurocognitive database from the years 1994–2008. At our department all patients receive a comprehensive standard-of-care neuropsychological assessment (including the domains intelligence, processing speed, visuospatial processing, attention, memory, executive functioning, visuomotor functioning, adaptive behavior and quality of life) at the time of diagnosis and at predefined time points after diagnosis of CNS-cancer (depending on tumor type and treatment modality: at least after therapy and at 1–3 year intervals in aftercare). Assessment results were documented in the above mentioned database. For this study, only data regarding the domains intelligence, processing speed and visuomotor functioning were analysed. All patients included in this study were tested with the German version of one of the WS. Depending on age and point of assessment the Wechsler Intelligence Scale for Children [21, 22], or the Wechsler Adult Intelligence Scale was applied [23]. All versions of the WS consist of a set of verbal subtests in order to collect information on VIQ and a set of performance subtests to collect information on PIQ. Finally, FIQ as an indicator of overall intellectual ability can be calculated. Even if—for clinical use—only FIQ, VIQ and PIQ should be interpreted, for this study, a detailed subtest analysis was performed. Other indices, e.g. the working memory index or the processing speed index, which can be calculated in newer versions of the WS, did not exist then. For further comparative analyses the scores of the different WS subtests were merged into one variable for each domain. The Trailmaking Test-Form A (TMT-A) was used in order to investigate information processing speed and visuomotor function [24]. TMT-A requires a person to connect circled numbers in chronological order while time is measured.

### Data collection

For this retrospective study we analysed data from the neurocognitive database of 62 consecutive patients, who were diagnosed and treated for MB between the years 1992 and 2008 at our institution. Before 1994 neurocognitive data were scarcely available, after 2008 other neurocognitive tests were used that take the speed–power problem at least partially into account, e.g. the newer version of the WS [25]. Due to missing data (because of death, changing residency or a health condition in which a neuropsychological evaluation was not possible) 25 patients had to be excluded, but for a total number of 37 consecutive patients information for more than one timepoint was available.

We retrospectively analysed four timepoints of assessment: after surgery (up to 4 months postoperative; for 12 patients WS, for seven TMT-A and for six both tests were available), 1 year after surgery (for 11 patients WS, for 11 TMT-A and for eight both tests were available), 2 years after surgery (for seven patients WS, for seven TMT-A and for four both tests were available) and 3 years after surgery (for ten patients WS, for 7 TMT-A and for five both tests were available). For the given reasons, not all 37 patients underwent neurocognitive assessment at all four timepoints (for detailed information see Online Resource 1).

### Sample characteristics

Out of n = 37 consecutive patients with MB, 26 were male (70.3%) and 11 female (29.7%). The age at diagnosis ranged between 3.1 and 21.6 years with a mean age of 9.8 years (SD = 3.97 years), time since diagnosis ranged between 0 and 3 years. All patients were treated with a combination of surgery, chemo- and radiotherapy according to the applicable treatment protocols, depending on the year of diagnosis (for detailed information see Online Resource 1).

### Statistical methods

The statistical analysis was conducted using IBM SPSS^®^ Statistics Version 24. Results were interpreted at a significance level of 5%. To answer the hypothesis that the subtest mean values of children with MB differ from the norm, one sample *t*-tests were computed and the effects were expressed in units of standard deviation (Cohen’s d). Small effects were classified at a level of 0.2, medium effects at 0.5 and large effects at 0.8 [26]. Results are shown for each timepoint of assessment. To test the influence of processing speed/visuomotor performance, an independent sample *t*-test was computed (for further details see Online Resource 2). This analysis was done cross-sectionally, since it can be assumed that the influence on IQ-scores is stable over time. For a more detailed analysis, TMT-A scores were correlated with the single subtests of the WS for the time point 1 year after diagnosis. This assessment date was chosen, since—from clinical experience—illness- and treatment-related effects are likely to be visible already, but patients in the 1990s and early twenty-first century rarely had a neurocognitive training at that stage of the illness, which might have influenced the outcome. As correlation coefficient Kendall’s τ was used, since it is less influenced by rank ties [27]. Correlations of τ = ±0.1 were regarded as small, τ = ±0.3 as medium and τ = ±0.5 as large effects [28].

## Results

Regarding our first research question, whether IQ-scores in pediatric patients with MB differ significantly from the mean value of the standardization sample (µ = 100, σ = 15), we found that FIQ of the patients was significantly lower 1 year after surgery (mean FIQ = 86.64, SD = 16.77, t = −2.643, df = 10, p = 0.025, d = −0.89). However, no difference for the assessment timepoint up to 4 months after surgery could be observed (mean FIQ = 98.58, SD = 20.13, t = −0.244, df = 11, p = 0.812, d = −0.09). At the later time points, no significant but nevertheless relevant effects could be found for FIQ. Looking at PIQ, mostly large and also significant effects were found, again especially for the time point 1 year after surgery (mean PIQ = 80.18, SD = 17.50, t = −3.755, df = 10, p = 0.004, d = −1.27). For VIQ no significant deviation from the norm could be found at any time point of assessment (cf. the detailed results in Table 1).

**Table 1:** Deviation of FIQ, PIQ and VIQ from the norm

	Date of assessment (years after surgery)	Mean	SD	t	df	Sig.^a^	Cohen’s d^b^
FIQ	0	98.58	20.13	−0.244	11	0.812	−0.09
	1	86.64	16.77	−2.643	10	0.025*	−0.89
	2	87.71	20.94	−1.552	6	0.172	−0.82
	3	89.80	21.23	−1.519	9	0.163	−0.68
PIQ	0	86.91	17.07	−2.543	10	0.029*	−0.87
	1	80.18	17.50	−3.755	10	0.004*	−1.27
	2	89.57	16.34	−1.689	6	0.142	−0.7
	3	83.25	21.44	−2.209	7	0.063	−1.12
VIQ	0	105.18	18.95	0.907	10	0.386	0.35
	1	95.82	13.67	−1.015	10	0.334	−0.28
	2	91.00	22.72	−0.970	5	0.376	−0.60
	3	96.63	24.63	−0.388	7	0.710	−0.22

^a^One sample *t* tests, asterisks* mark significant results (p < 0.05)

^b^Cohen’s d illustrates the deviation from the standardized mean of the norm group (µ = 100, σ = 15) in units of SD

In a cross-sectional paired samples *t*-test, VIQ and PIQ turned out to differ significantly from each other for the first two time points (up to 4 months after surgery: mean difference = −18.273, SD = 12.338, t = −4.912, df = 10, p = 0.001; 1 year after surgery: mean difference = −15.636, SD = 10.433, t = −4.971, df = 10, p = 0.001).

**Fig. 1:**
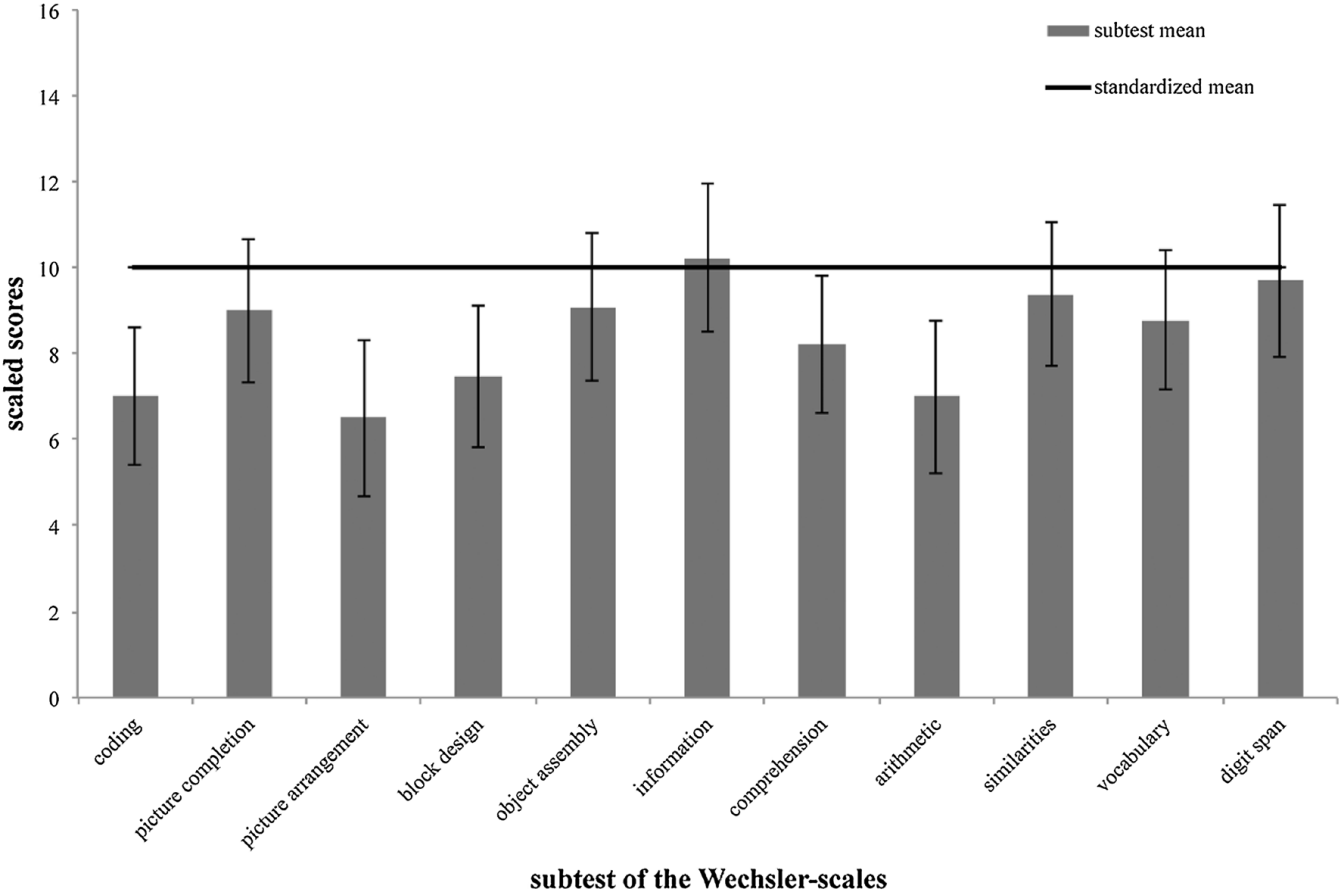
Deviation of the mean subtest performance from the norm value 1 year after surgery (scaled scores, standardized mean = 10, SD = 3)

Looking at a detailed subtest analysis (standardized subtest means = 10, standardized subtests SD = 3), we observed that at the time point 1 year after surgery three out of five subtests composing PIQ turned out to show a significant mean deviation from the norm (coding: mean score = 6.91, SD = 2.66, t = −3.850, p = 0.003, d = −1.03; picture arrangement: mean score = 6.50, SD = 3.41, t = −3.745, p = 0.003, d = −1.17; block design: mean score = 7.33, SD = 2.77, t = −3.330, p = 0.007, d = −0.89), while for the VIQ-subtests this was only true for the arithmetic subtest (mean score = 6.92, SD = 3.29, t = −3.249, p = 0.008, d = −1.03), which has—like no other verbal subtest but like each performance subtest—a speed component. Tables 2 and 3 give an overview of the detailed results regarding the subtests composing PIQ and VIQ, depending on time of assessment. Moreover, for the assessments 1 year after surgery, Fig. 1 shows the detailed subtest scores in relation to the standardized mean.

**Table 2:** Deviation of the PIQ-subtests from the norm

PIQ-subtest	Date of assessment (years after surgery)	Mean	SD	t	df	Sig.^a^	Cohen’s d^b^
Coding	0	7.62	3.23	−2.663	12	0.021*	−0.79
	1	6.91	2.66	−3.850	10	0.003*	−1.03
	2	6.50	2.62	−3.780	7	0.007*	−1.17
	3	7.00	3.07	−3.245	10	0.009*	−1.00
Picture completion	0	12.00	3.80	1.291	5	0.253	0.67
	1^c^	9.00	0.82	−2.449	3	0.092	−0.33
	3	11.50	4.36	0.688	3	0.541	0.50
Picture arrangement	0	6.29	2.70	−5.145	13	0.000*	−1.24
	1	6.50	3.41	−3.745	12	0.003*	−1.17
	2	8.71	3.73	−0.912	6	0.397	−0.43
	3	7.56	3.47	−2.115	8	0.067	−0.81
Block design	0	9.07	2.87	−1.211	13	0.247	−0.31
	1	7.33	2.77	−3.330	11	0.007*	−0.89
	2	7.63	2.13	−3.148	7	0.016*	−0.79
	3	7.90	3.87	−1.715	9	0.120	−0.70
Object assembly	0	9.50	3.15	−0.550	11	0.593	−0.17
	1	9.08	3.15	−1.009	11	0.335	−0.31
	2	9.57	2.64	−0.430	6	0.682	−0.14
	3	8.13	3.76	−1.411	7	0.201	−0.62

^a^One sample *t* tests, asterisks* mark significant results (p < 0.05)

^b^Cohen’s d illustrates the deviation from the standardized mean of the norm group (µ = 10, σ = 3) in units of SD

^c^This subtest was not administered at time point 2 years after diagnosis

**Table 3:** Deviation of the VIQ-subtests from the norm

VIQ-subtest	Date of assessment (years after surgery)	Mean	SD	t	df	Sig.^a^	Cohen’s d^b^
Information	0	10.67	3.24	0.796	14	0.439	0.22
	1	10.08	3.09	0.093	11	0.927	0.03
	2	9.25	4.20	−0.505	7	0.629	−0.25
	3	9.45	3.30	−0.549	10	0.595	−0.18
Comprehension	0	10.00	3.13	0.000	9	1.000	0.00
	1	8.00	2.65	−2.268	8	0.053	−0.66
	2	8.63	3.29	−1.181	7	0.276	−0.46
	3	9.14	4.30	−0.528	6	0.617	−0.29
Arithmetic	0	8.50	3.96	−1.419	13	0.180	−0.50
	1	6.92	3.29	−3.249	11	0.008*	−1.03
	2	7.86	3.93	−1.441	6	0.200	−0.71
	3	8.60	4.22	−1.049	9	0.322	−0.47
Similarities	0	11.77	2.95	2.164	12	0.051	0.59
	1	9.17	2.86	−1.011	11	0.334	−0.28
	2	9.38	2.88	−0.615	7	0.558	−0.21
	3	11.09	3.67	0.985	10	0.348	0.36
Vocabulary	0	11.70	4.75	1.285	12	0.223	0.57
	1	9.00	2.63	−1.318	11	0.214	−0.33
	2	8.63	3.34	−1.166	7	0.282	−0.45
	3	9.00	3.63	−0.913	10	0.383	−0.33
Digit span	0	9.50	3.70	−0.506	13	0.621	−0.17
	1	9.83	3.22	−0.180	11	0.861	−0.06
	2	7.43	2.82	−2.413	6	0.052	−0.86
	3	8.55	3.64	−1.324	10	0.215	−0.48

^a^One sample *t* tests, asterisks* mark significant results (p < 0.05)

^b^Cohen’s d illustrates the deviation from the standardized mean of the norm group (µ = 10, σ = 3) in units of SD

Our second research question was, whether there is a significant difference in IQ-scores between patients with at least average processing speed/visuomotor function scores and patients with a performance below average. We presumed that the group with no impairment shows higher FIQ and PIQ, but equal VIQ scores, since processing speed/visuomotor function is predominantly inherent in the PIQ. Furthermore, we presumed that the correlation between TMT-A scores and the performance part of the WS is significant and higher than between TMT-A and the verbal part of the WS. We expected a substantial correlation only between TMT-A and the VIQ-subtest “arithmetic”, as this subtest also underlies a speed component.

According to the results, patients with no processing speed/visuomotor function impairment showed higher FIQ scores (FIQ mean = 101.75, SD = 14.02) than patients with an impairment (FIQ mean = 87.50, SD = 11.11). This result was significant (t = −2.408, df = 18, p = 0.027). The same is true for PIQ. Again, patients with no impairment showed higher scores (PIQ mean = 98.27, SD = 12.56) than patients having a slower processing speed/worse visuomotor function (PIQ mean = 79.00, SD = 12.75, t = −3.281, df = 17, p = 0.004). On the contrary, regarding VIQ no significant difference could be observed (VIQ mean = 95.50 vs. 103.92, SD = 11.52 vs. 16.99, t = −1.221, df = 18, p = 0.238).

Finally, processing speed seemed to impact PIQ, but not VIQ. In order to further analyse the correlations between processing speed/visuomotor function and the WS, we correlated TMT-A and the WS subtests at the time point 1 year after surgery. Table 4 shows the detailed results. As can be seen, there was a moderate correlation between TMT-A and FIQ (τ = 0.294, p = 0.232). Looking at this result in more detail it revealed a zero-correlation between TMT-A and VIQ and a fairly large and also significant correlation between TMT-A and PIQ (τ = 0.689, p = 0.005). Moreover, a detailed subtest analysis showed especially high and also significant, correlations between TMT-A and the subtests coding (τ = 0.606, p = 0.004) and object assembly (τ = 0.556, p = 0.018). Within the VIQ-subtests, only small or moderate correlations could be found, all of them lacking significance (including the subtest “arithmetic”).

**Table 4:** Correlation between TMT-A and WS 1 year after diagnosis

Correlation TMT-A+PIQ	Kendall’s τ^a^	Sig.*	Correlation TMT-A+VIQ	Kendall’s τ^a^	Sig.*
PIQ	0.689	0.005*	VIQ	<0.001	>0.999
Coding	0.606	0.004*	Information	0.404	0.221
Picture completion	–^b^	–^b^	Comprehension	0.320	0.343
Picture arrangement	−0.250	0.414	Arithmetic	0.313	0.543
Block design	0.243	0.546	Similarities	0.294	0.232
Object assembly	0.556	0.018*	Vocabulary	0.92	0.535
TMT-A+FIQ	0.294	0.232	Digit span	0.404	0.221

*Significant results (p < 0.05)

^a^For the use of Kendall’s τ as an indicator of the correlation see above in the methods section

^b^Due to missing data the correlation coefficient could not be estimated

## Discussion

The overall objective of this retrospective study was to discuss the usefulness of the IQ-concept for the description of neurocognitive outcome of pediatric CNS-tumor patients. We found strong support for our main hypothesis that FIQ cannot be interpreted independently from certain underlying neurocognitive functions, especially information processing speed and visuomotor function. This finding underlines the call for using a detailed neuropsychological assessment instead of sole g-factor interpretations of intelligence like FIQ [29], due to the mixture of the speed- and power-component in the WS, like in many other intelligence test-batteries, in order to avoid misinterpretations of neurocognitive outcome.

Even though newer versions of the WS (and other intelligence test-batteries) take the speed component into account to a certain extent (e.g. by calculating separate processing speed indices), the fact that FIQ is influenced by processing speed still remains (since arithmetic and PIQ-subtests still have an inherent speed component). Interpreting IQ-scores exclusively therefore has probably already led to a remarkable misinterpretation of cognitive outcome of CNS-tumor patients. Especially pediatric patients with MB are at high risk for such misleading studies, since patients with MB are more prone towards a reduction of processing speed and visuomotor function due to tumor location and irradiation treatment [3, 5].

Fortunately, newer studies—unlike older ones—sometimes already use larger neurocognitive test batteries instead of single outcome measures [16]. Given the present results and taking the guidelines into account, such an approach has to be endorsed [7, 8]. Often, however, institutions worldwide consider the IQ as a popular and fairly easy-to-interpret score and are, moreover, lacking the resources needed for a detailed assessment. Therefore, future longitudinal studies should focus on the costs of overseeing long-term effects and compare these costs to the expenses for additional resources. At least, a stepwise assessment procedure should be adopted in all institutions with a broad screening of neurocognitive functions and a more detailed assessment in cases where certain risk factors can be identified.

By analysing the neuropsychological profile of a patient we are able to identify those parameters which may lead to a lower academic achievement and participation restrictions. By using FIQ only, particularly school counselling is at high risk of wrong decision making with negative effects on the general academic achievement of these children: if FIQ is further used as the only measure of general performance, one may conclude that a child needs a lower-level curriculum; however if the child only processes information at a slower pace (which is the case for a large number of patients as shown in this study), but logical thinking is intact, as is often the case in patients with MB, one may only ask for extra time during exams.

In addition, the present study showed that the impact of information processing speed and visuomotor function might be different at various time points after diagnosis for pediatric patients with MB. Even though the results have to be interpreted with caution (since it is a retrospective study and longitudinal data were not available for all patients), we can hypothesize that the most dramatic change occurs within the first year after surgery, i.e. during treatment, which is consistent with existing literature [12–16]. At this point of assessment, in our cross-sectional design, subtests with a speed-component turned out to have the lowest scores. Later on, in the course of the disease, the cognitive profile—of course depending on the underlying neurocognitive functions—seems to reach at least a certain stability, if not improvement. This contrasts—to some extent—previous findings [30–32], where a continuous decline was reported. This is possibly due to the different focus of these studies, either on very young age at diagnosis or on molecular subgroups or on long-term outcome [30–32]. Future studies should therefore analyse the influence of processing speed decline on intellectual outcome in more detail with respect to these specific patient subgroups. Especially research on the outcome of the different molecular MB subgroups seems promising with respect to processing speed decline.

Nevertheless, the FIQ is not appropriate for a detailed consideration of neurocognitive functions, as it does not give any sophisticated information about the strengths and deficits of children. Furthermore, regarding intelligence tests, often a conflation of processing speed or visuomotor function and FIQ can be found (e.g. quicker patients score higher in a visuospatial subtest). Even though newer versions of the WS calculate a separate processing speed index, a confounding of processing speed and other functions can still be found. Taking these arguments into account, it is—as suggested by the Children´s Oncology Group guidelines– the better strategy to administer an IQ test plus a detailed neurocognitive test battery instead of over-interpreting the FIQ [7, 8]. This is especially true for domain-specific interventions in pediatric neurooncology. Some even suggest measuring cognitive outcome exclusively with neuropsychological tests, as they offer more detailed information about the way a child learns, administrates and organizes [29]. Future research on MB outcome, especially regarding molecular subgroups, should therefore also rely on distinct neurocognitive testing.

In summary, the present study appeals to the professionals in the area of neurocognitive outcome after pediatric CNS-cancer to use a detailed neurocognitive profile analysis rather than global outcome measures like the FIQ in order to interpret cognitive outcome. In the past, the overestimation of the rather uninformative FIQ may have led to a faulty evaluation of the cognitive abilities of pediatric patients with MB, since in FIQ-tests processing speed is often confounded with other domains. Hence, distinct measures for each domain should be used. Despite objective difficulties in achieving developmental milestones, some patients possibly may have shown difficulties just because of such underestimations and subsequent wrong counselling.

Apart from the already mentioned retrospective and not fully longitudinal character of this study, some further limitations have to be mentioned. Despite the homogeneous group of patients, the sample size was small: multicenter studies may reveal further insight into the relationship between information processing speed and overall intelligence in the future. Moreover, missing data turned out to further diminish the sample size and made multivariate analyses hardly possible, which shows the necessity of more prospective studies in this field. Furthermore, no significant but still relevant effects were found regarding the relationship between VIQ and information processing speed. This may be to a certain extent due to the fact that one VIQ-subtest—arithmethic—also has a speed-component. In addition, many patients were absent from school for treatment-related reasons, which could also explain the medium correlation between processing speed and information (i.e. general knowledge, vocabulary, arithmetic). But again, this shows the necessity of interpreting a differentiated profile of neurocognitive functions. Consequently, the value of this study lies in a re-thinking of outcome measures and in highlighting the possibilities of using a larger neurocognitive test battery instead of solely interpreting global outcome measures like the IQ.

## Electronic supplementary material

Below is the link to the electronic supplementary material.

Supplementary material 1 (DOCX 20 KB)

Supplementary material 2 (DOCX 14 KB)
