# Supplementary material for: Do we still need IQ-scores? Misleading interpretations of neurocognitive outcome in pediatric patients with medulloblastoma: a retrospective study
Source: J Neurooncol. 2017 Aug 4;135(2):361–9. doi: 10.1007/s11060-017-2582-x (PMC5663794; doi:10.1007/s11060-017-2582-x)
Supplement: Supplementary file 1 — Supplementary material 1 (DOCX 20 KB) [file 11060_2017_2582_MOESM1_ESM.docx]

**Online Resource 1: Individual patient characteristics**

| Patient | sex | age at onset  y;m | treatment protocol | M ^a)^ | H^b)^ | CMS ^c)^ | death | Neurocogn. assessment |
| --- | --- | --- | --- | --- | --- | --- | --- | --- |
| 1 | m | 13;7 | HIT 91 sandwich | M0 | no signs | n/a ^d)^ | - | + |
| 2 | m | 5;10 | HIT 91 sandwich | n/a | n/a ^d)^ | n/a ^d)^ | × | D ^f)^ |
| 3 | m | 8;3 | HIT 91 sandwich | M0 | no signs | n/a ^d)^ | - | + |
| 4 | f | 4;4 | HIT 91 sandwich | M0 | n/a ^d)^ | n/a ^d)^ | LFU ^e)^ | + |
| 5 | m | 7;4 | HIT 91 sandwich | M0 | no signs | n/a ^d)^ | - | + |
| 6 | m | 20;0 | n/a ^d)^ | n/a ^d)^ | n/a ^d)^ | n/a ^d)^ | LFU ^e)^ | LFU ^e)^ |
| 7 | m | 14;0 | HIT 91 sandwich | M0 | no signs | n/a ^d)^ | LFU ^e)^ | + |
| 8 | f | 3;8 | n/a ^d)^ | n/a ^d)^ | n/a ^d)^ | n/a ^d)^ | - | PS ^g)^ |
| 9 | m | 13;3 | HIT 91 sandwich | M3 | no signs | n/a ^d)^ | × | + |
| 10 | f | 5;4 | HIT 91 maintenance | M0 | VP-Shunt | n/a ^d)^ | - | + |
| 11 | f | 7;1 | HIT 91 maintenance | M0 | VP-Shunt | n/a ^d)^ | - | + |
| 12 | m | 11;0 | HIT 91 sandwich | M0 | VP-Shunt | n/a ^d)^ | - | + |
| 13 | f | 15;2 | HIT 91 maintenance | M0 | VP-Shunt | n/a ^d)^ | - | LFU ^e)^ |
| 14 | m | 9;5 | HIT 91 sandwich | M0 | VP-Shunt | n/a ^d)^ | × | + |
| 15 | m | 14;9 | HIT 91 sandwich | n/a ^d)^ | VP-Shunt | n/a ^d)^ | × | LFU ^e)^ |
| 16 | m | 19;4 | n/a ^d)^ | n/a ^d)^ | n/a ^d)^ | n/a ^d)^ | × | LFU ^e)^ |
| 17 | m | 15;6 | HIT 91 sandwich | M4 | VP-Shunt | yes | × | + |
| 18 | m | 7;8 | HIT 91 maintenance | M0 | EVD | n/a ^d)^ | - | + |
| 19 | f | 11;5 | other | n/a ^d)^ | VP-Shunt | n/a ^d)^ | × | LFU ^e)^ |
| 20 | m | 12;4 | HIT 91 sandwich | M0 | no signs | n/a ^d)^ | - | + |
| 21 | f | n/a ^d)^ | n/a ^d)^ | n/a ^d)^ | n/a ^d)^ | n/a ^d)^ | LFU ^e)^ | LFU ^e)^ |
| 22 | f | 3;10 | HIT 91 sandwich | M2 | EVD | n/a ^d)^ | - | PS ^g)^ |
| 23 | m | 12;5 | HIT 91 sandwich | M4 | no signs | n/a ^d)^ | - | + |
| 24 | m | 3;6 | HIT 91 maintenance | M0 | EVD | n/a ^d)^ | - | + |
| 25 | m | 10;7 | n/a ^d)^ | n/a ^d)^ | n/a ^d)^ | n/a ^d)^ | LFU ^e)^ | + |
| 26 | m | 12;1 | HIT 2000 | M0 | VP-Shunt | n/a ^d)^ | - | + |
| 27 | f | 4;0 | HIT 91 sandwich | M0 | EVD | n/a ^d)^ | - | PS ^g)^ |
| 28 | m | 1;8 | HIT 91-SKK | M0 | Yes | no | - | PS ^g)^ |
| 29 | f | 4;8 | HIT 91 sandwich | M0 | VP-Shunt | yes | - | PS ^g)^, language |
| 30 | f | 4;1 | HIT 91 sandwich | M3 | no signs | n/a ^d)^ | × | PS ^g)^ |
| 31 | f | 6;5 | HIT 91 sandwich | M0 | no signs | n/a ^d)^ | × | + |
| 32 | m | 15;2 | other | M0 | no signs | n/a ^d)^ | - | + |
| 33 | m | 12;0 | HIT 91 sandwich | M0 | no signs | n/a ^d)^ | - | + |
| 34 | m | 12;11 | HIT 91 maintenance | n/a ^d)^ | no signs | n/a ^d)^ | × | physically bad condition |
| 35 | m | 9;8 | HIT 91 maintenance | M0 | no signs | n/a ^d)^ | × |  |
| 36 | f | 2;2 | HIT 91 SKK | M4 | VP-Shunt | n/a ^d)^ | × | PS ^g)^ |
| 37 | f | 9;2 | HIT 2000 | n/a ^d)^ | no signs | n/a ^d)^ | - | + |
| 38 | m | 1;4 | HIT 91 SKK | M0 | EVD, ETV | yes | × | PS ^g)^ |
| 39 | f | 21;6 | other | M0 | no signs | no | - | + |
| 40 | f | 8;7 | HIT 2000 | M3 | n/a ^d)^ | n/a ^d)^ | - | + |
| 41 | m | 5;2 | HIT 2000 | M2 | no signs | no | - | + |
| 42 | m | 5;7 | HIT 2000 SKK | M1 | VP-Shunt | yes | × | + |
| 43 | m | 9;2 | HIT 91 sandwich | M0 | EVD | n/a ^d)^ | × | + |
| 44 | m | 10;3 | HIT 2000 SKK | M1 | VP-Shunt | n/a ^d)^ | - | + |
| 45 | f | 8;1 | HIT 2000 | M0 | EVD | no | - | + |
| 46 | f | 3;1 | HIT 200 SKK | M0 | ETV | no | - | + |
| 47 | m | 4;11 | HIT 2000 | n/a ^d)^ | EVD | no | LFU ^e)^ | language |
| 48 | m | 10;3 | HIT 91 sandwich | M1 | no signs | yes | - | + |
| 49 | m | 9;9 | HIT 2000 | n/a ^d)^ | EVD | no | - | + |
| 50 | m | 8;4 | HIT 2000 | n/a ^d)^ | no signs | no | - | + |
| 51 | f | 8;1 | HIT 91 sandwich | n/a ^d)^ | EVD | yes | - | + |
| 52 | f | 4;0 | HIT 2000 SKK | M1 | VP-Shunt | yes | - | + |
| 53 | m | 16;6 | HIT 2000 | n/a ^d)^ | EVD | n/a ^d)^ | - | + |
| 54 | m | 11;9 | HIT 2000 SKK | n/a ^d)^ | EVD, ETV | yes | × | + |
| 55 | m | 9;11 | other | n/a ^d)^ | no signs | n/a ^d)^ | × | language |
| 56 | m | 15;2 | HIT 2000 | M0 | no signs | no | × | physically bad condition |
| 57 | f | 3;2 | HIT 2000 SKK | M0 | no signs | no | - | PS ^g)^ |
| 58 | m | 11;7 | HIT 2000 | M4 | EVD, ETV | n/a ^d)^ | - | + |
| 59 | m | 5;9 | HIT 2000 | M1 | VP-Shunt | yes | × | language |
| 60 | f | 6;6 | other | n/a ^d)^ | VP-Shunt | n/a ^d)^ | LFU ^e)^ | language |
| 61 | m | 3;7 | HIT 2000 SKK | n/a ^d)^ | EVD | yes | - | PS ^g)^ |
| 62 | m | 1;3 | HIT 2000 SKK | M0 | EVD | no | - | PS ^g)^ |

*Note:* ^a)^ metastases at diagnosis ^b)^ hydrocephalus and surgical intervention (EVD = external ventricular drainage, ETV = endoscopic third ventriculostomy, VP-Shunt = ventriculo-peritoneal shunting) ^c)^ cerebellar mutism syndrome (before 2001 data were not recorded systematically, therefore a large amount of data was not available) ^d)^ data not available ^e)^ lost to follow up ^f)^ different test battery ^g)^ pre-school age
