# Supplementary material for: Do we still need IQ-scores? Misleading interpretations of neurocognitive outcome in pediatric patients with medulloblastoma: a retrospective study
Source: J Neurooncol. 2017 Aug 4;135(2):361–9. doi: 10.1007/s11060-017-2582-x (PMC5663794; doi:10.1007/s11060-017-2582-x)
Supplement: Supplementary file 2 — Supplementary material 2 (DOCX 14 KB) [file 11060_2017_2582_MOESM2_ESM.docx]

**Online Resource 2: Processing of TMT-A data**

The Trailmaking Test A [1] requires a person to connect circled numbers in chronological order on a sheet of paper while time is measured. The manual provides cut-off scores. According to these the performance of a patient (overall time) can be divided into „below average“, „average“ and „above average“. Newer Versions of the Trailmaking Test (e.g. in the Delis-Kaplan Executive Function System, 2001) also provide standardized scores for the time needed for circling the numbers in the correct order.

The results of TMT-A were also used to group patients depending on their processing speed / visuomotor function scores into patients with a performance below average and into patients with an at least average performance.

Since TMT-A in the applied form only results in ordinally scaled variables (below, within or above average range), data from the WS were also transformed into ordinally scaled variables (again with three possible values), using ± 1 SD as average range (as proposed by the test and by a European convention).

The following table shows the descriptive statistics of the Trailmaking Test A in our sample:

Table 1: descriptive statistics for processing speed of medulloblastoma patients in the TMT-A for the different assessment time points.

|  | date of TMT-A assessment (years after surgery) | | | |
| --- | --- | --- | --- | --- |
|  | 0  (n=7) | 1  (n=11) | 2  (n=7) | 3  (n=7) |
| TMT-A-score below average | 42.9% | 36.4% | 57.1% | 42.9% |
| average TMT-A score | 28.6% | 54.5% | 42.9% | 57.1% |
| TMT-A score above average | 28.6% | 9.1% | 0% | 0% |

[1] Reitan RM (1959) A manual for the administration and scoring of the Trailmaking-Test. Indian University Medical Center, Indianapolis
